# Human 3D Lung Cancer Tissue Photothermal Therapy Using Zn- and Co-Doped Magnetite Nanoparticles

**DOI:** 10.1021/acsbiomaterials.4c01901

**Published:** 2025-01-24

**Authors:** Edynara Cruz de Moraes, Marcella Miranda Siqueira Furtuoso Rodrigues, Rafaela Campos de Menezes, Marcus Vinícius-Araújo, Marize Campos Valadares, Andris Figueiroa Bakuzis

**Affiliations:** † Institute of Physics, Federal University of Goiás, Goiânia, Goiás 74690-900, Brazil; ‡ ToxIn-Laboratory of Education and Research in In Vitro Toxicology, Federal University of Goiás, Goiânia 74690-631, Brazil; § CNanoMed, Federal University of Goiás, Goiânia, Goiás 74690-631, Brazil

**Keywords:** iron oxide, thermal-induced immunotherapy, nanomedicine, cytokine, reactive oxygen species

## Abstract

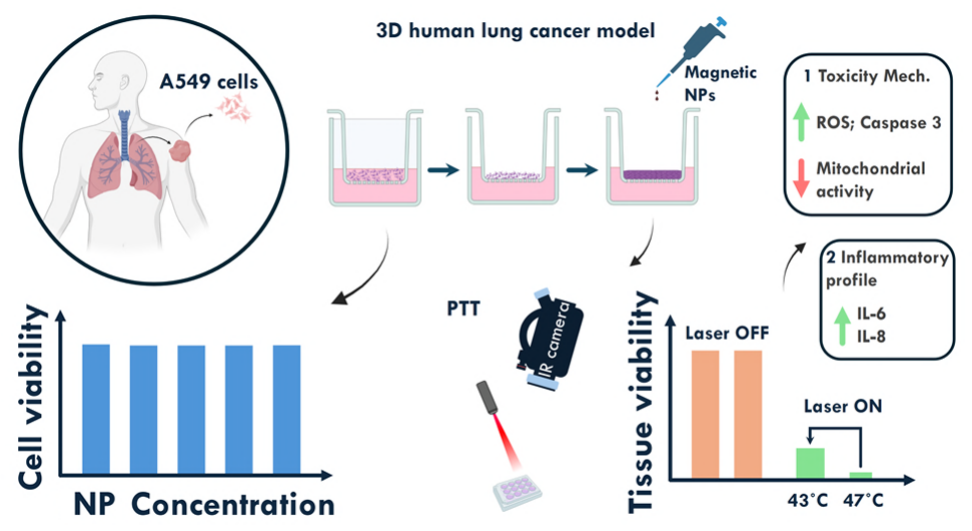

Iron oxide-based nanoparticles are promising materials for cancer thermal therapy and immunotherapy. However, several proofs of concept reported data with murine tumor models that might have limitations for clinical translation. Magnetite is nowadays the most popular nanomaterial, but doping with distinct ions can enhance thermal therapy, namely, magnetic nanoparticle hyperthermia (MNH) and photothermal therapy (PTT). In this study, we used a 3D alveolar reconstructed A549 lung cancer tissue model and investigated the thermal properties, toxicity, and impact of the thermal dose on tissue viability and inflammatory response using magnetite codoped with 40% Zn and 2% Co divalent ions. The ZnCo-doped magnetite nanoparticles are not toxic up to an NP concentration of 30 mg/mL. PTT showed a better heat generation response than MNH under the evaluated conditions, while NP showed a high external photothermal conversion efficiency of ∼1.3 g·L^–1^·cm^–1^ at 808 nm. PTT study is carried out at different temperatures, 43 and 47 °C, for 15 min. Tissue viability decreased with increasing thermal dose, while intracelullar ROS levels increased, mitochondrial activity decreased, and active caspase-3 increased, suggesting cell death via apoptosis. Nanoparticles and PTT did not influence the cytokine TNF, IL-10, IL-1B, and IL-12p70. In contrast, IL-6 and IL-8 were triggered by NP and PTT. Increased expression of IL-6 and IL-8 with higher thermal doses is correlated with tissue injury results, suggesting the potential role in activating and attracting immune cells to the site of thermal-mediated tissue injury.

## 1 Introduction

Iron oxide-based nanoparticles (NPs) are promising materials for thermal therapies. NPs generate heat under AC magnetic field stimulation, a phenomenon known as magnetic nanoparticle hyperthermia (MNH), or through light absorption in the near-infrared region (NIR), called photothermal therapy (PTT).^1−3^ Heat can promote cell death, with important applications for cancer therapy.^4−6^ In addition, when the NPs biodegrade, the release of metallic ions results in the activation of several immunological responses in mouse models. For example, it tunes macrophage polarization to the M1-like state, resulting in a delay in tumor growth and a decrease in the focus of metastases.^7^ On the other hand, thermal therapies are believed to be key players for both innate and adaptive immune cells.^4^ However, most proofs of concept for cancer therapy are performed in murine models that could hamper clinical translation.

Alternative human cancer models have been suggested in the literature.^8,9^ For example, patient-derived xenograft (PDX) mouse models are becoming more popular, but there are concerns. In most cases, tumors are implanted under the skin of the mouse and not on the relevant tissue-specific support (orthotopic site). The stromal component of the tissue could be replaced by mouse-derived stroma.^8^ Mice with humanized immune cell lines have been developed, but differences in human response have been reported.^8^ Human cell culture strategies are very common, but this traditional 2D model also has limitations since it does not reflect the tissue architecture or the real cell–cell and cell–tissue interaction scenario. Therefore, there is a pressing need to evaluate cancer therapies using more representative human-like cancer models. This is particularly crucial for diseases such as lung cancer, which remains the leading cause of death worldwide.

A promising approach to improve the relevance of in vitro studies is the use of 3D reconstructed tissue models. In this study, we opt for the 3D alveolar reconstructed A549 lung cancer model, which incorporates an air–liquid interface cell system to closely mimic the microenvironment of the human lung.^10^ Compared with traditional 2D models, the 3D reconstructed A549 lung cancer model offers a more accurate representation of the in vivo lung environment. By simulation of respiratory conditions, this model provides physiologically relevant outcomes. In addition, it facilitates improved cell–cell interactions, improved drug testing, and the ability to culture long-term, making it an attractive platform to study lung cancer and assess potential therapies.^11^

In a previous study,^10^ the group cultivated on an air–liquid interface a 3D alveolar epithelial model and, more importantly, morphologically characterized it by demonstrating the expression of several biomarkers with human alveoli. Cell cohesion was demonstrated by the regular expression of CD44, which was evident in the intracelullar junctions. The epithelial phenotype of alveolar cells was investigated by evaluating the expression of Pan-cytokeraton and MUC-1, which were related to mucus production. Only E-cadherin presented a different expression, particularly at the basal layers of the epithelium. The results clearly prove the relevance of this alveolar 3D reconstructed tissue model to mimic the human scenario.

In recent years, traditional A549 cell culture models have served as vital tools to investigate the cytotoxic effects of various NPs and their potential applications in thermal therapy.^12−15^ Lin et al. explored the impact of silica NPs on human A549 lung cancer cells, observing a dose-dependent reduction in cell viability attributed to increased oxidative stress.^16^ Similarly, Simon-Deckers et al. examined the biological responses of A549 cells to titanium oxide and carbon nanotubes, highlighting the varying toxicities of different nanomaterials.^17^ Choi et al. explored the cytotoxic effects of metal hydroxide NPs on A549 cells, revealing pronounced inflammatory responses and oxidative stress at high concentrations.^18^

Until now, the presented studies have predominantly explored 2D models, which inherently possess limitations, as discussed. However, one notable exception used a 3D A549 cell tissue model. Moacă et al. investigated the biological impact of magnetic iron oxide NPs on lung cancer cell lines A549 and NCI-H460 using EpiAirway 3D in vitro microtissues to assess biosafety profiles and cytotoxic effects.^19^ The investigation revealed a variable cytotoxic effect of magnetic iron oxide NPs on A549 and NCI-H460 lung cancer cell lines but without exploring cell death mechanisms. The AlamarBLUE test demonstrated a more pronounced impact on the viability of A549 cells compared with NCI-H460 cells, suggesting a higher sensitivity of A549 cells to NPs.

Despite this, there are no studies using PTT as a stress agent in 3D alveolar reconstructed A549 lung cancer. In particular, a traditional 2D A549 cell culture has been explored for NP-based thermal therapy, particularly using magnetic hyperthermia. For instance, Dutta et al. explored the potential of curcumin-loaded gelatin-grafted Fe_3_O_4_ magnetic NPs in hyperthermia combined therapy for A549 cells, demonstrating enhanced cytotoxicity under an alternating magnetic field.^20^ Furthermore, Kim et al. developed magnetic nanoparticle-conjugated polymeric micelles for the treatment of A549 cancer cells, revealing significant decreases in cell proliferation when combined with magnetic hyperthermia.^21^ Whang et al. introduced a synergistic therapy that combines magnetic hyperthermia treatment and radiation therapy using zinc–manganese ferrite magnetic NPs, highlighting enhanced apoptotic effects in A549 cells through active targeting and combination therapy strategies.^22^ Collectively, these 2D-like studies underscore the potential of NP-based thermal therapies in the context of A549 lung cancer treatment.

From the wide range of magnetic NPs that have been used in A549 cell culture cytotoxicity studies, iron oxide variants are the most prevalent. In this article, we explore the codoping of magnetite with Zn and Co using a hydrothermal method, as this type of nanomaterial has shown significant potential to improve magnetic properties and increase the efficiency of heat delivery. Zn doping enhances the magnetization of the NPs, while Co doping can tune the magnetic anisotropy, which is crucial for improving heat generation. Previous studies have demonstrated that the combination of codoping with Zn and Co allows simultaneous enhancement of both magnetothermal and photothermal responses.^23,24^ Furthermore, we were also inspired by the potential immunological response arising from the release of metallic ions due to the possibility of NP biodegradation. According to the literature, some metal ions such as Fe and Zn play a major role in metalloimmunology.^25^ Zn-based nanomaterials have been reported to increase the response of CD8^+^ T cells, reduce immune-suppressive Treg cells, and enhance T cell infiltration, among other factors.^25^ However, cobalt has been less studied and has been of greater concern regarding toxicity. Co is a necessary component of vitamin B12, is important as a coenzyme of cell mitosis, and might be relevant for the treatment of l-Arg auxotrophic tumors.^26,27^ Recent studies suggest that at low and moderate concentrations, Co induces an M2-like phenotype in macrophages, while at high concentrations and long-term stimulations, it increases the expression of TLR4 and promotes the M1-like phenotype.^28^ Fe plays a crucial role in macrophage polarization toward the M1-like phenotype, resulting in a decrease in metastases and long survival in murine tumor models.^7,25^ Furthermore, heat can trigger innate and adaptive immune cells.^4^ So, combining NPs with thermal therapy might improve patient treatment.

In this study, magnetite was synthesized and codoped with 40% Zn and 2% Co divalent ions, and the properties of MNH and PTT were compared to magnetite NP. We demonstrate that PTT is more efficient than MNH in generating heat under the conditions employed, particularly for the ZnCo-doped NPs. We report the photothermal conversion efficiency of the NPs and choose the best one, ZnCo-doped iron oxide NPs, to investigate their cytotoxicity within the 3D alveolar epithelium model. Tissue viability under different conditions, namely, temperatures of 43 °C and 47 °C, indicates that cell death depends on the thermal dose. Inflammatory responses have also been reported to investigate how thermal therapy triggers the release of cytokines. Possible implications for heat-triggered immunotherapy are briefly discussed.

## 2 Materials and Methods

### 2.1 Synthesis of Magnetic NPs

The following commercially available chemical reagents were used without further purification: ZnCl_2_·4H_2_O, CoCl_2_·4H_2_O, FeCl_2_·4H_2_O, FeCl_3_·6H_2_O, and methylamine (CH_3_NH_2_) all purchased from Sigma-Aldrich (St. Louis, Missouri, USA). All solutions were prepared using Milli-Q water.

The synthesis of Fe_3_O_4_ and Zn_0.4_Co_0.02_Fe_2.58_O_4_ NPs was performed by using a hydrothermal method. First, standard solutions containing Fe^3+^ (1 M), Fe^2+^ (1 M), Zn^2+^ (1 M), and Co^2+^ (1 M) ions were prepared. Then, to produce the magnetite (Fe_3_O_4_), appropriate amounts of Fe^3+^ and Fe^2+^ were combined, followed by the addition of 10 mL of methylamine (CH_3_NH_2_) diluted in 40 mL of DI water, resulting in the formation of a black precipitate. This precipitate was autoclaved at 80 °C for a duration of 4 h. After the reaction, it was washed three times with Milli-Q water and prepared for citrate coating (Na_3_C_6_H_5_O_7_) to obtain a stable colloid. Furthermore, the codoping of magnetite with zinc (Zn) and cobalt (Co) ions, with concentrations of 40% for Zn and 2% for Co, was explored. The quantity of Fe^2+^ was adjusted to match the concentration of the respective dopant ions.

### 2.2 Photothermal Conversion Efficiency (PCE)

The photothermal conversion efficiency (PCE) can be determined using eq 1:
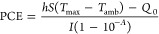
1

In this equation, the variables are defined as follows: *h* denotes the heat transfer coefficient, *S* represents the surface area of the container, *T*_max_ is the equilibrium temperature, *T*_amb_ is the ambient temperature, *Q*_0_ characterizes the heat absorption of the Eppendorf tube, *I* stands for the laser power, and *A* corresponds to the absorbance of the NPs at a wavelength of 808 nm.

Furthermore, the product of *hS* can be reasonably estimated when the system reaches a steady equilibrium state, as indicated by eq 2:
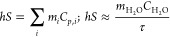
2

Subsequently, during the cooling phase, the following relationship was applied to assess the temporal evolution:
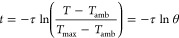
3

Hence, the time constant τ can be accurately determined through a linear fit by using the aforementioned equation.

Additionally, Paściak et al.^29^ introduced recently a new parameter to assess the heating performance, which is defined as ePCE = *a*_λ_PCE. In this expression, *a*_λ_ is given by , where *A*_λ_ represents the absorbance at a specific wavelength, *c* is the mass concentration (mg/mL), and *L* is the optical path (cm).^29^

### 2.3 X-ray Diffraction

X-ray diffraction measurements of powder samples were performed using a Bruker D8 Discover X-ray diffractometer, which operated with Cu Kα radiation at a wavelength of 0.15 nm. The diffraction data were collected in the angular range of 2θ spanning 10 to 90° using a step size of 0.01 degrees. It should be noted that the instrument in question is located at the CRTI (Regional Center for Technological Development and Innovation) facility at the University of Goiás (UFG).

### 2.4 Cell Culture

The Nutrient Mixture F-12 Ham medium (HAM-F12), fetal bovine serum (FBS), and trypan blue dye were obtained from Sigma-Aldrich, while penicillin/streptomycin and TrypLE Express Trypsin/EDTA were purchased from Gibco.

The A549 human cell line, originally described by Lieber et al. in 1976,^30^ was obtained from the Banco de Células do Rio de Janeiro, located in Rio de Janeiro, Brazil. These cells were cultured in Nutrient Mixture F-12 Ham medium (HAM-F12) and supplemented with 10% fetal bovine serum (FBS) along with 1% penicillin/streptomycin. The culture environment was meticulously maintained under conditions of 5% CO_2_ in a humidified atmosphere at 37 °C, with medium changes carried out at intervals of 2 to 3 days. When reaching a confluence of 80–90%, cells were rinsed with PBS followed by detachment using TrypLE Express Trypsin/EDTA. To determine cell viability, the trypan blue dye exclusion method (0.2%) was used. Cell counting was performed using the automated cell counter TC20, a product of Bio-Rad. Experimental procedures were initiated exclusively when cell viability exceeded the 90% threshold.

### 2.5 Assessment of Cell Viability

To establish the concentration of NPs for further investigations, the effects of different concentrations of NP on the monolayer of A549 cells were evaluated. The cells were seeded at a density of 5 × 10^5^ cells/well in a total volume of 100 μL in a 96-well plate. After a 24 h incubation period, the cells were exposed to NP concentrations of 5, 10, 15, 20, 25, and 30 mg/mL, or HAM-F12 medium as a control. Cell viability was evaluated using the trypan blue dye exclusion method 24 h after exposure to the NPs.

### 2.6 Alveolar 3D Epithelial Model

For the alveolar epithelium model, a Type I Collagen matrix (BD Biosciences, NJ, USA) was coated onto 24-well transwell inserts with 0.4 μm diameter pores (Falcon, Tewksbury, MA, USA). Collagen Type I (BD Biosciences, NJ, USA) was mixed with 1 M NaOH, 10 M concentrated DMEM medium, and water. A total of 200 μL of the mixture was added to the transwell inserts and incubated at 37 °C for 15 min, after which the gel was washed twice with 100 μL of PBS. A549 cells were seeded at 5 × 10^5^ cells per insert and incubated for 5 h. The apical medium was then carefully removed to create an air–liquid interface. The medium in the basal compartment of the culture system contained HAM-F12 medium, 10% FBS, and 1% penicillin/streptomycin and was maintained at 37 °C in a humidified atmosphere of 5% CO_2_/95% air. The assembly of the model was considered as day 0, and it was analyzed until day 3. NP treatment was performed on day 3 for 3 h, followed by laser exposure for 15 min. The tissues were then washed three times with PBS, fixed in 4% paraformaldehyde (pH 7.4), and embedded in OCT medium (Tissue-Tek, Sakura, Radnor, PA, USA). Once embedded, 5 μm slices were prepared and stained with hematoxylin/eosin to evaluate the structural organization. The evaluation and photodocumentation were conducted using an optical microscope (DM 2000, Leica Microsystems, Bannockburn, USA).

### 2.7 Exposure of NPs to the 3D Alveolar Epithelium Model

NPs were dispersed in phosphate-buffered saline (PBS) at a concentration of 10 mg/mL. Subsequently, apical exposures were carried out employing 100 μL of the NP suspension, and tissues were cultured for 3 or 24 h at 37.0 °C in a 5.0% CO_2_ environment. Temperature exposures of 43 °C and 47 °C were applied for 15 min and monitored using an IR camera FLIR SC620 vertically positioned. For irradiation, an 808 nm laser was utilized, located at a distance of 43 cm from the model. Following irradiation, the apical surface of the tissues was washed three times with PBS.

### 2.8 Assessment of Tissue Viability

The cytotoxic potential of the NPs was assessed in the 3D alveolar epithelium model using the MTT (3-(4,5-dimethylthiazol-2-yl)-2,5-diphenyltetrazolium) assay. The tissue was incubated with 600 μL per base well of HAM-F12 medium containing a 0.5 mg/mL MTT solution for 3 h at 37 °C. The resulting crystals were dissolved in 1 mL of isopropanol under agitation at 30 rpm for 3 h. Optical density measurements were performed at 560 nm using a plate spectrophotometer (Multiskan Spectrum, Thermo Scientific, Waltham, MA, USA).

### 2.9 Reactive Oxygen Species (ROS) Assay

After exposures, the reconstructed tissues were removed from the insets and incubated with a 50 μM DCFH-DA (Sigma-Aldrich, St. Louis, MO, USA) reagent for 30 min. The tissues were washed three times with PBS and transferred to molds containing Tissue-Tek OCT medium (Sakura, Radnor, PA, USA) for further freezing in liquid nitrogen. Posteriorly, the models were cryosectioned into 5 μm slices, transferred to microscopy slides, and evaluated by fluorescence microscopy (DMI 4000 B, Leica Microsystems, Bannockburn, USA) coupled to the software LAS-AF, filter L5.

### 2.10 Mitochondrial Activity Assay

For the evaluation of mitochondrial damage, tissues were incubated with a 200 nM MitoTracker reagent for 1 h. The tissues were washed three times with PBS, transferred to molds containing an OCT medium, and frozen in liquid nitrogen. Posteriorly, the models were cryosectioned into 5 μm slices, transferred to microscope slides, and evaluated by fluorescence microscopy (DMI 4000 B, Leica Microsystems, Bannockburn, USA) coupled to the software LAS-AF, filter N1.

### 2.11 Activity of Caspase

The expression of caspase in tissues was evaluated via indirect immunofluorescence. The tissues were embedded in an OCT medium (Tissue-Tek, Sakura, Radnor, PA, USA), rapidly frozen in liquid nitrogen, and sectioned into thin slices using a Cryostat (CM 1850, Leica Microsystems, EUA). The slides were incubated overnight in a humid chamber with the primary anti-Active Caspase-3 Monoclonal Antibody 1:100 (Cell Signaling Technology, MA, USA). After that, the slides were washed 4 times with PBS and incubated for 1 h with the secondary antibody antimouse AlexaFluor 488 1:500 (Invitrogen, CA, USA). Nuclear staining was performed with Hoechst dye (5 ng/mL) for 5 min. Lastly, the slides were washed four times, mounted, and evaluated in fluorescence microscopy (DMI 4000 B, Leica Microsystems, Bannockburn, USA) coupled to the LAS-AF software, filter A4 (blue), filter N21 (red), and filter N21.

### 2.12 Tissue TEM Image

For TEM analyses, the material was first fixed in Karnovsky solution containing 4% paraformaldehyde and 2.5% glutaraldehyde in sodium phosphate for 24 h, followed by immersion in 1% osmium tetroxide for 2 h. After fixation, the material was dehydrated using an increasing series of acetones and subsequently embedded in an Epon resin. The ultrathin sections were then contrasted with 2% uranyl acetate and 0.2% lead citrate for visualization under a Jeol JEM-2100 transmission electron microscope at the UFG Laboratory of High-Resolution Microscopy (LabMic).

### 2.13 Flow Cytometer Analysis

After 4 h of exposure, cytokine levels in the basal and apical medium were quantified using the BD CBA Human Inflammatory Cytokines Kit (BD Biosciences, San Jose, CA, USA). This kit facilitated the quantitative measurement of interleukin-8 (IL-8), interleukin-1β (IL-1β), interleukin-6 (IL-6), interleukin-10 (IL-10), tumor necrosis factor (TNF), and interleukin-12p70 (IL-12p70) protein levels within a single sample. The procedures were performed according to the manufacturer’s instructions. Sample measurements were performed using a BD FACS Canto II Flow Cytometer and analyzed using the FCAP Array software (BD Biosciences).

### 2.14 Statistical Analysis

The results were expressed as the mean value ± standard deviation of the values obtained from various replicates of the experiments. Intergroup variations were evaluated using a one-way ANOVA multiple comparison test, with statistical significance defined at *p* < 0.05. All analyses were performed using the GraphPad Prism software version 8.0 (San Diego, CA, USA).

## 3 Results and Discussion

### 3.1 Structural and Morphological Characterization of NPs

**Figure 1:**
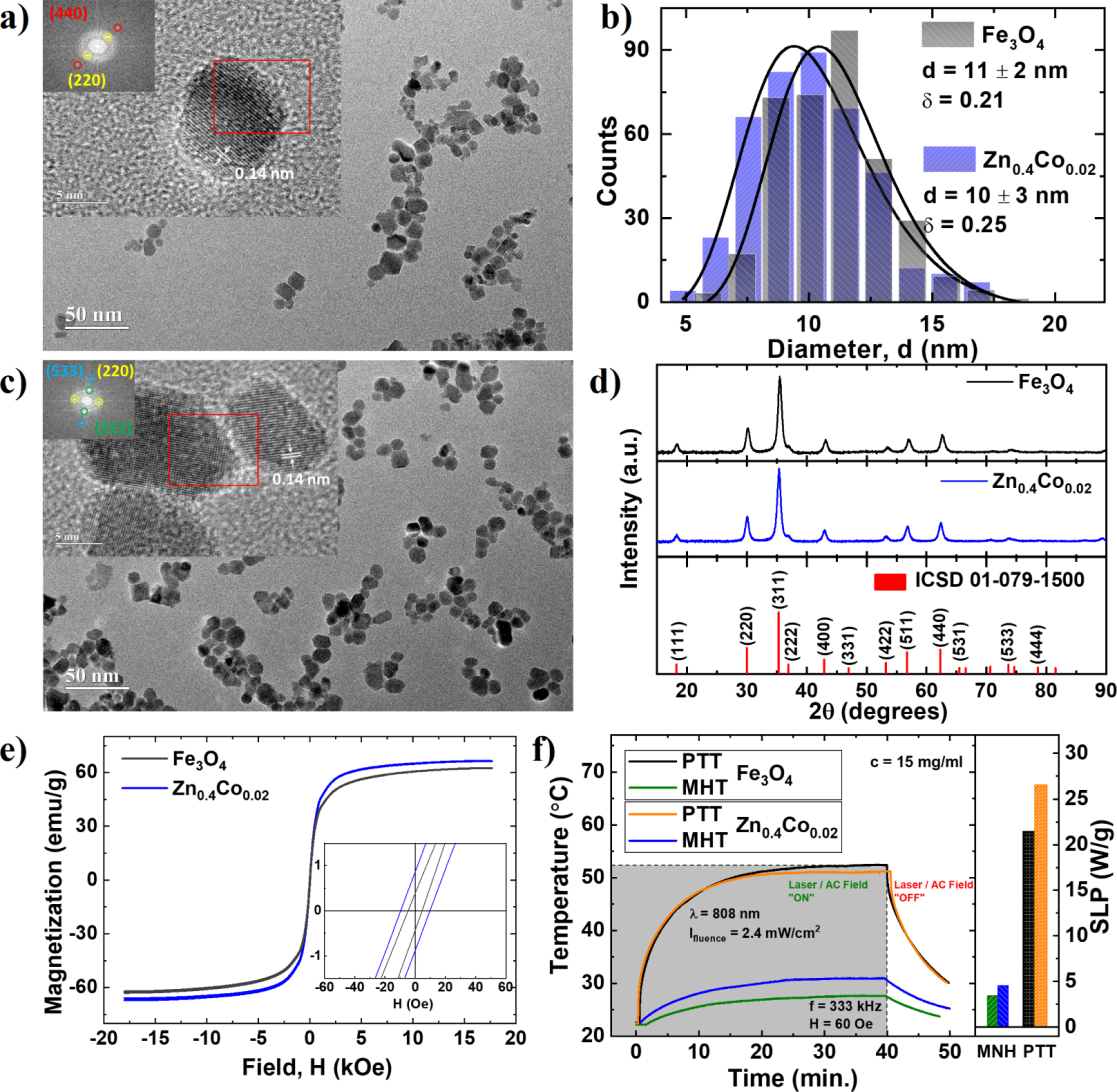
TEM and HRTEM (inset) images for samples (a) Fe_3_O_4_ and (b) Zn_0.4_Co_0.02_ samples. (c) Log-normal size distribution of the Fe_3_O_4_ and Zn_0.4_Co_0.02_ NPs. (d) Powder X-ray diffraction data. (e) Room temperature magnetization for samples Fe_3_O_4_ and Zn_0.4_Co_0.02_. (f) Variation in temperature over time for PTT and MNH, and SLP for Fe_3_O_4_ and Zn_0.4_Co_0.02_ samples at 808 nm.

Figure 1a,c shows transmission electron microscopy (TEM) images and high-resolution TEM (HRTEM) images in the inset for Fe_3_O_4_ and Zn_0.4_Co_0.02_Fe_2.58_O_4_ (Zn_0.4_Co_0.02_) NPs. The images reveal the predominant presence of spherical-shaped NPs. Additionally, the insets of Figure 1a,c showcase HRTEM images and their corresponding fast Fourier transform (FFT) analyses, elucidating the distinct planes and orientations of the inverse spinel magnetite. Specifically, FFT analyses revealed interatomic distances of 0.28 nm (220) and 0.14 nm (440) in Figure 1a, while Figure 1c displays interatomic distances of 0.28 nm (220), 0.23 nm (222), and 0.12 nm (533). Furthermore, TEM images facilitated determination of the log-normal size distribution of the NPs, as illustrated in Figure 1b. From this distribution, the mean diameter of the NPs was determined to be  nm for the Fe_3_O_4_ sample and  nm for the Zn_0.4_Co_0.02_ sample. Figure 1d shows the XRD patterns of the Fe_3_O_4_ and Zn_0.4_Co_0.2_ samples. The results confirm the spinel structure of the NPs, as evidenced by the standard magnetite structure cataloged by ICSD 01-079-1500.

### 3.2 Magnetic and Photothermal Properties

Magnetic evaluation was carried out at room temperature for the Fe_3_O_4_ and Zn_0.4_Co_0.02_ samples (Figure 1e). Incorporation of Zn and Co in the structure of the nanomaterial is observed to increase the *M*_s_ value (saturation magnetization). An increase in the *H*_c_ (coercive field) is observed, which confirms the presence of Co within the structure. The determined specific saturation magnetization was found to be 60.6 emu/g for Fe_3_O_4_ and 68.1 emu/g for Zn_0.4_Co_0.02_ NPs. The heating properties of the synthesized NPs were investigated at a concentration of 15 mg/mL using samples with a volume of 500 μL of magnetic fluid. For PTT, the experimental parameters comprised a laser power of 200 mW with a wavelength (λ) of 808 nm, accompanied by an estimated laser spot size of 0.084 cm^2^, corresponding to a fluence of 2.4 W/cm^2^. Regarding MNH, the experiment was conducted at a frequency of 333 kHz and an applied field of 60 Oe. To achieve thermal equilibrium within the system, an irradiation time of 40 min was used. Temperature variation measurements of the samples were continuously monitored in real-time using an IR FLIR SC620 camera. Figure 1f illustrates the temporal evolution of the temperature for both methodologies, where PTT exhibited superior heat delivery, achieving a temperature of approximately 52.5 °C for Fe_3_O_4_ and of approximately 50 °C for Zn_0.4_Co_0.02_, although a higher initial heat rate is observed for this sample. This observation is further supported by the substantial disparity in the calculated specific loss power (SLP) between the two techniques, as shown in Figure 1f. Particularly for iron-oxide NPs, previous studies have also shown that light-to-heat conversion under near-infrared excitation (PTT) performs better than heat generation stimulated by alternate magnetic field (MNH).^24,31^

Therefore, PTT over MNH and Zn_0.4_Co_0.02_ over Fe_3_O_4_ were selected for experiments with the human A549 cell line based on the significant difference observed in heat delivery efficiency between the two techniques, as evidenced by the superior temperature elevation achieved with PTT. In addition, PTT was chosen because of the possibility of heat being delivered to very small regions, allowing the treatment of local tumors/metastases, which could induce an abscopal effect.^32^ Furthermore, this choice was made to ensure optimal conditions to effectively assess the response of A549 cells to thermal stress.

**Figure 2:**
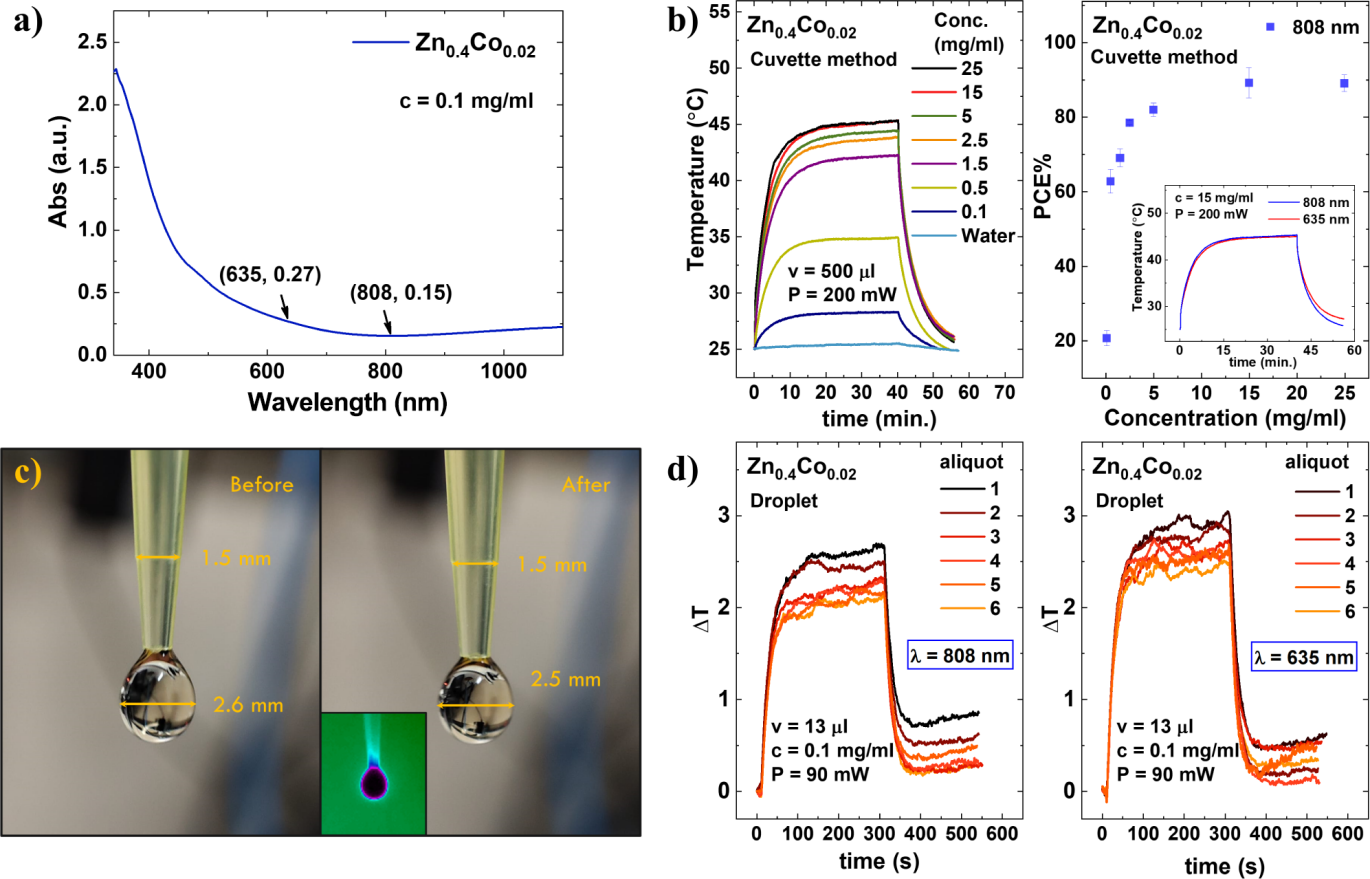
(a) Absorption measurement of the Zn_0.4_Co_0.02_ sample at 0.1 mg/mL concentration. (b) Heating profile at 808 nm at different concentrations of aliquot using the standard cuvette method and PCE in function of sample concentration. (c) Representative photo and thermal photo of a 13 μL droplet before and after the experiment. (d) Heating profile at 808 and 635 nm of several droplet aliquots at 0.1 mg/mL concentration and 13 μL volume.

To further analyze the photothermal efficiency of the Zn_0.4_Co_0.02_ sample, experiments were conducted using two laser wavelengths, 808 and 635 nm, and two distinct experimental approaches, namely, the cuvette and droplet methods. Figure 2a shows the absorption curve for this sample, which shows the typical decrease up to around 800 nm and a slight increase for higher wavelengths that appears in some studies of magnetite NPs.^33^ Both PCE and ePCE were calculated for each wavelength using Roper’s equation. The study used two different experimental setups: the cuvette method (Figure 2b) and the droplet method (Figure 2c,d). Using the cuvette method, with a volume of 500 μL and a NP concentration of 15 mg/mL, the PCE at 635 nm was determined to be 68%, while at 808 nm, the PCE increased to 89% considering the cooling regime. These PCE values are comparable to those reported for γ-Fe_2_O_3_.^29^ Notably, the PCE is concentration-dependent, as illustrated in Figure 2b at 808 nm, with lower values observed at lower concentrations and a saturation effect at higher concentrations. Similar heating profiles are observed for both wavelengths (see the inset for the comparison at 15 mg/mL concentration). Recently, Paściak et al.^29^ suggested the use of a droplet method and introduced the external photothermal efficiency parameter ePCE = *a*_λ_PCE, as described in the Materials and Methods, to provide a more consistent comparison of heating performance between materials. Here, , where *A*_λ_ represents the absorbance at a specific wavelength, *c* is the mass concentration (mg/mL), and *L* is the optical path (cm). The argument is that the droplet method minimizes issues related to the NP concentration and sample holder absorption effects. However, this method has limitations, such as evaporation, which can alter the droplet’s mass and NPs’ concentration during measurement. To avoid these effects, low particle concentrations and low laser power conditions are needed. In this analysis, we used the NP concentration of 0.1 mg/mL and a laser power of 90 mW. Figure 2c presents a representative photo of a 13 μL droplet before and after the experiment, showing a 0.1 mm decrease in the droplet diameter, equivalent to 4% of the droplet volume. At this concentration, we found a PCE value for the droplet method of 21%, which is the same value obtained for the cuvette method. For ePCE calculations, measurements were performed using different aliquots, as shown in Figure 2d. From the data in Figure 2d, the temperature variation at 808 nm was determined to be 2.4 ± 0.2 K, while at 635 nm, it was 2.8 ± 0.2 K. This outcome aligns with the observation that droplet temperature variation fluctuates within a certain range due to the low NP concentration and environmental conditions. Note that after cooling, the stationary temperature achieved differs from the initial condition. Additionally, the temperature variation, although not significant, could be attributed to varying environmental conditions in the setup room. For a 13 μL droplet, the ePCE was found to be 1.9 ± 0.3 g·L^–1^·cm^–1^ at 635 nm and 1.3 ± 0.2 g·L^–1^·cm^–1^ at 808 nm. These ePCE values obtained from the droplet method are comparable, but higher than those reported for γ-Fe_2_O_3_.^29^

### 3.3 Nanoparticle-Mediated PTT Effects on the 3D Human Tissue Lung Cancer Model

Before investigating the effect of NP interaction with reconstructed human tissue, preliminary cytotoxic studies were conducted using A549 cultured in a monolayer to establish work concentrations. As shown in Figure S2, the NPs did not promote cell death in all concentrations studied after 24 h of exposure. The cell viability was observed at particle concentrations up to 30 mg/mL. The results revealed no significant toxic effects on the cultured cells after a 24 h incubation period. We conclude that codoping the iron oxide NPs with Zn and Co, in the present amount, does not affect cell viability.

**Figure 3:**
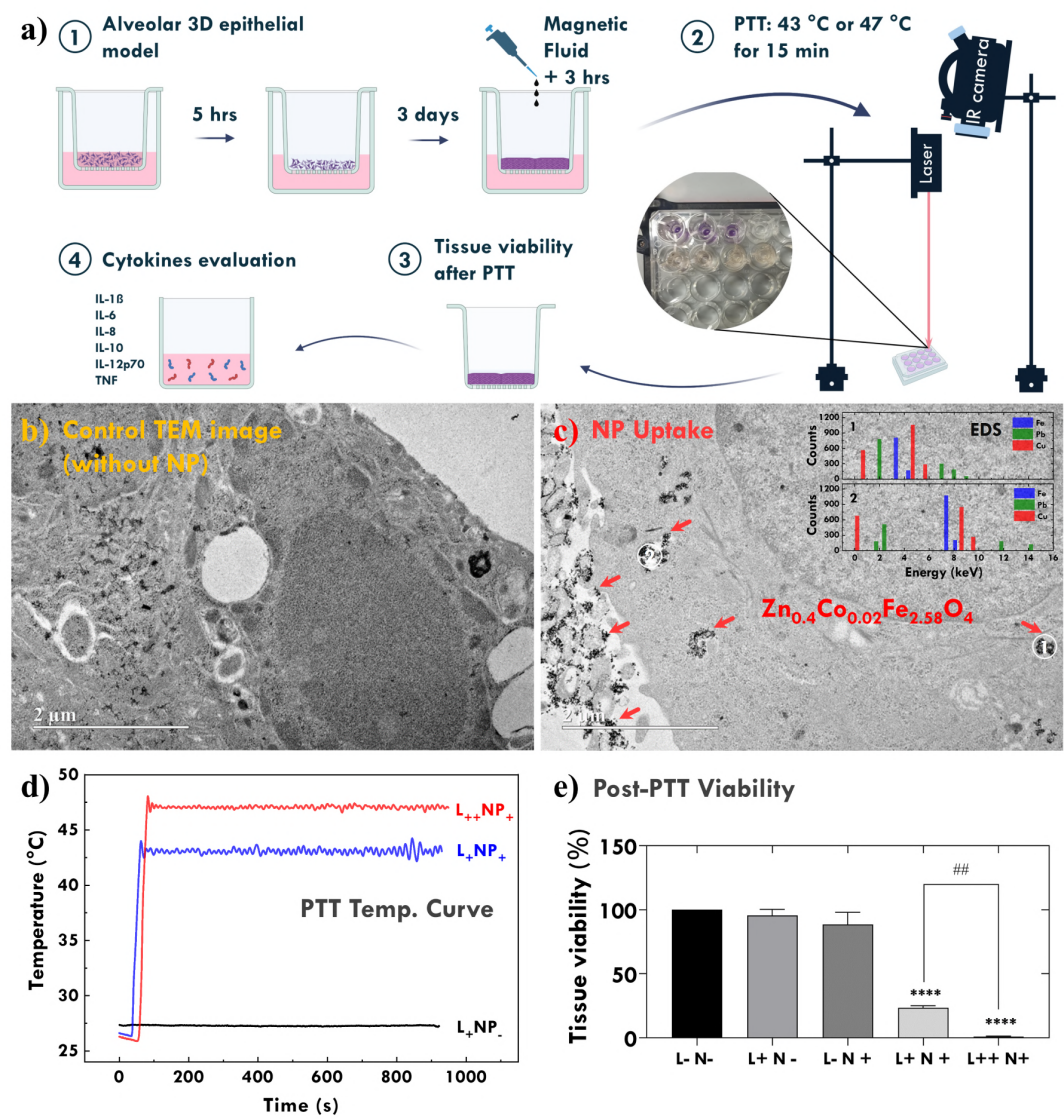
(a) Schematic of the photothermal therapy experiment; TEM images showing internalization of Zn_0.4_Co_0.02_Fe_2.58_O_4_-based NPs by cells in the 3D tissue. (b) Control group (without NP) and (c) tissue incubated with NPs prior to laser irradiation. Numerous NP aggregates are observed within the cell. EDS in regions 1 and 2 are shown in the inset of panel c. (d) Heating curves for temperatures of 43 ° and 47 °C. Abbreviations: L_–_ (no laser), L_+_ (laser 43 °C), L_++_ (laser 47 °C), N_–_ (no NPs), and N_+_ (aliquot concentration: 10 mg/mL). (e) Evaluation of tissue viability using the 3D alveolar epithelial model by the MTT reduction assay (****p < 0.0001 compared to negative control. ^##^*p* < 0.0013 compared to L_+_N_+_ and L_++_ N_+_). Biological replicates: *n* = 3. Abbreviations: L_–_ (no laser), L_+_ (laser 43 °C), L_++_ (laser 47 °C), N_–_ (no NP), and N_+_ (aliquot concentration: 10 mg/mL).

Further tissue experiments were performed using a concentration of 10 mg/mL Zn_0.4_Co_0.02_ NPs. A representative image of the NP-mediated PTT study is shown in Figure 3a. After cultivating the 3D alveolar epithelial model for 3 days, followed by NP uptake by the tissue for an additional day, the tissue model was prepared for PTT experiments. The PTT was performed at a thermal dose of 43 °C or 47 °C for 15 min. Afterward, the tissue and basal medium were separated for tissue viability and cytokine release evaluation, respectively. Additionally, to evaluate whether NPs are internalized by cells in the 3D tissue, TEM images were acquired. Figure 3b is the control group (without NP), and Figure 3c is the tissue incubated with NP (before laser irradiation). Several aggregates of NPs are observed inside the cell. To confirm that these correspond to NPs, EDS was performed in two regions (1 and 2). The EDS analysis is shown in the inset of Figure 3c. Original EDS data can be found in Figure S1. The data confirm the presence of iron because of the NPs, that of Cu because of the TEM grid, and that of Pb because of the method of preparation of the sample for TEM analysis.

Figure 3d illustrates the temperature profiles over time during exposure of the model to temperatures of 43 and 47 °C. The thermal dose was carefully controlled by tuning the laser power to maintain the temperature established in the protocol. As shown in Figure 3e, it is evident that under all experimental conditions in which the NPs were subjected to laser exposure within the 3D alveolar epithelium model, there was a statistically significant reduction in tissue viability compared to the control group (*p* < 0.0001). Furthermore, a statistically significant distinction was observed when comparing laser exposures at 43 °C and 47 °C (*p* < 0.0013). However, when NPs or laser was administered individually, no reduction in tissue viability was observed. The laser alone, even at high power, does not cause significant heating, indicating that heat is only released when near-infrared radiation interacts with the electrons of the NPs, and through nonradiative transitions releases energy. The temperature profile shown in Figure 3d reflects the experimental procedure. Initially, the laser was applied at high power, resulting in a rapid temperature increase as the light interacts with the NPs. However, since the study focuses on investigating the role of thermal dose, once the critical temperature is reached, the laser power is adjusted to maintain the thermal dose throughout the PTT experiment.

The motivation to investigate distinct thermal doses arises from previous studies.^34−37^ It is well-known that at 43 °C, within the hyperthermia range, cells become more sensitive, leading to a higher death rate.^38^ On the other hand, heat-induced immunological responses have been the focus of recent research.^4^ For instance, using a bilateral tumor model and magnetic hyperthermia, authors have shown evidence of the abscopal effect and activation of CD8 cells within the hyperthermia range.^34,35^ Other studies, at higher temperatures, have revealed immunogenic cell death in the ablation regime.^36,37^ Currently, it remains unclear which temperature range is optimal for inducing an immunological response, and the effect may depend on the tumor cell type. Therefore, we chose to investigate two distinct thermal dose conditions.

**Figure 4:**
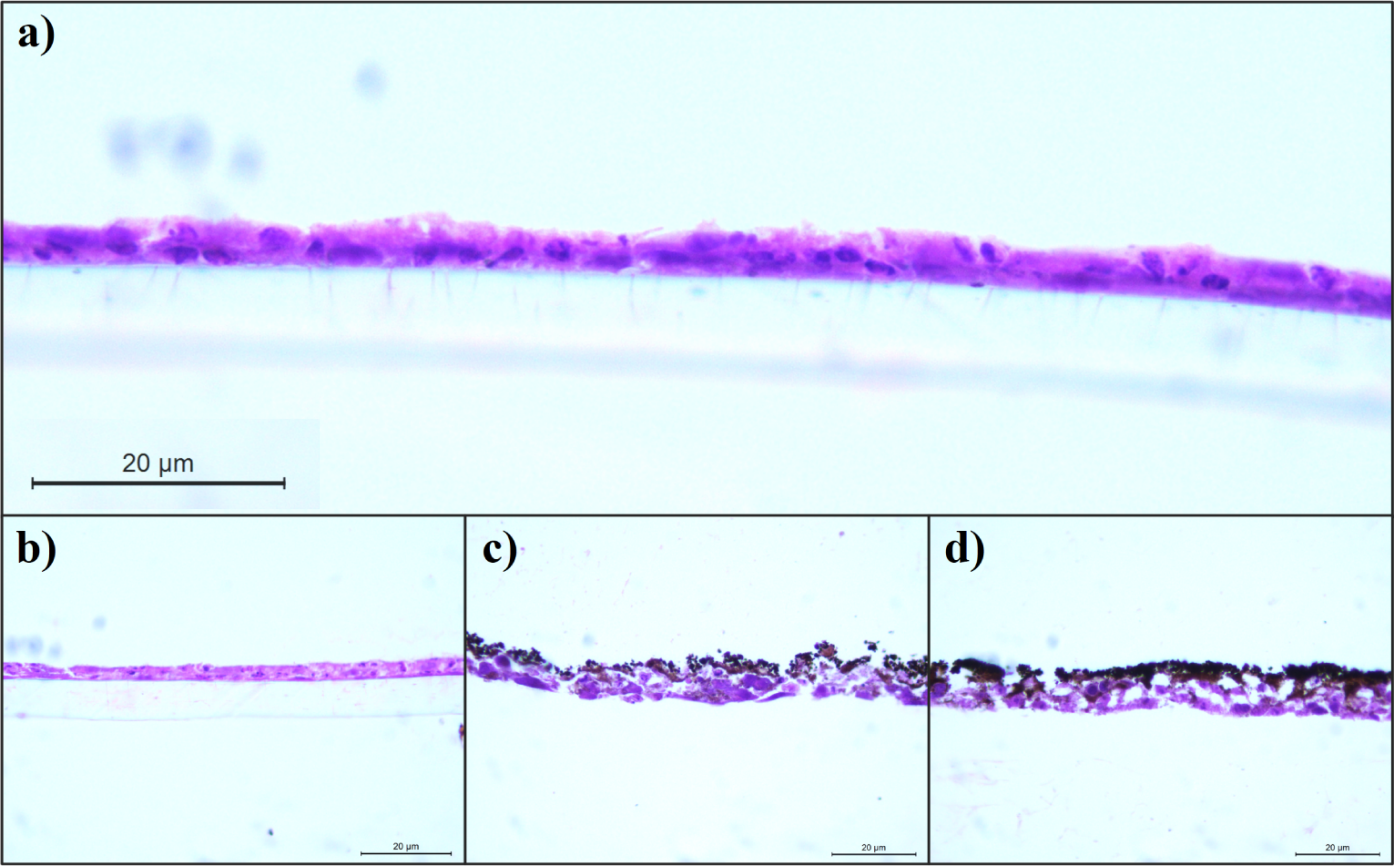
Photomicrograph showing the morphology of the 3D model assembled from human alveolar A549 cells on day 3 of culture. The images were obtained after staining the sections with hematoxylin/eosin and acquired using an optical microscope with a 40× objective lens. (a) Control group. (b) Tissue with laser irradiation only. (c) Tissue incubated with the Zn_0.4_Co_0.02_Fe_2.58_O_4_-based NPs prior to laser irradiation. (d) Tissue incubated with Zn_0.4_Co_0.02_Fe_2.58_O_4_-based NPs followed by 808 nm wavelength laser irradiation (aliquot concentration: 10 mg/mL).

**Figure 5:**
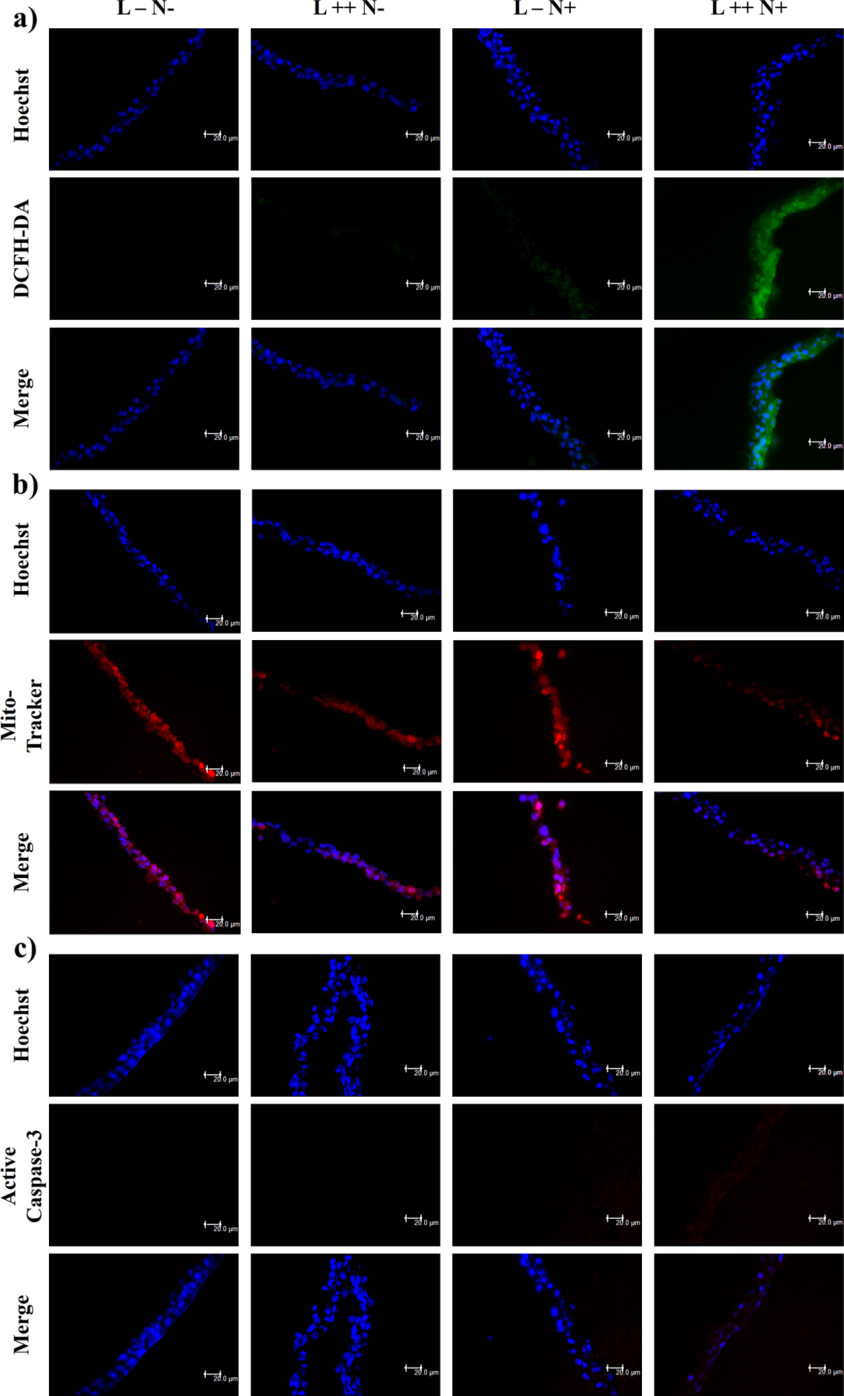
Assessment of toxicity mechanisms in a 3D model of A549 human alveolar cells after exposure. (a) Generation of reactive oxygen species (ROS) detected by DCFH-DA staining. (b) Mitochondrial activity evaluated in the 3D epithelial model using MitoTracker Red. (c) Expression of active caspase-3 identified through indirect immunofluorescence. All images were captured using a fluorescence microscope with a 40× objective lens. Abbreviations: L– (no laser), L++ (laser 47 °C), N– (no NP), N+ (NP at 10 mg/mL).

The tissue morphological analysis was also performed after exposure to NPs, laser alone, or both combined. Figure 4 shows photomicrographs of 3D human tissue under various experimental conditions. As expected, the nonexposed alveolar tissue displayed physiological morphology, and similar results were observed after exposure to the laser alone. In contrast, in the tissue exposed to both the laser and NPs, we observed signs of tissue destructuring with particle infiltration. However, the tissue exposed only to NPs appeared to show impregnation with the nanomaterial without significant structural changes. To further confirm particle infiltration, the micrographic images presented in Figure 4 were converted to binary images (Figure S3). The average pixel intensity corresponding to the NPs embedded in the tissue increased in the laser-exposed tissue compared to the nonirradiated tissue (see Figure S4). This indicates a higher degree of NP distribution embedded in the tissue. Additionally, studies were performed to investigate the mechanisms of cell death. As can be seen in Figure 5, exposure of tissue to laser + NPs increased intracellular ROS levels (Figure 5a) in the reconstructed tissue compared to NPs or laser alone. Moreover, in the laser + NPs’ group, we observed a reduction in mitochondrial activity compared to the other groups (Figure 5b), suggesting a potential effect of the NPs on alveolar cells. Furthermore, activation of the apoptosis pathway was detected in this group, as evidenced by the increased expression of active caspase-3 (Figure 5c) after exposure to NPs. Together, these results suggest cell death through apoptotic mechanisms.

**Figure 6:**
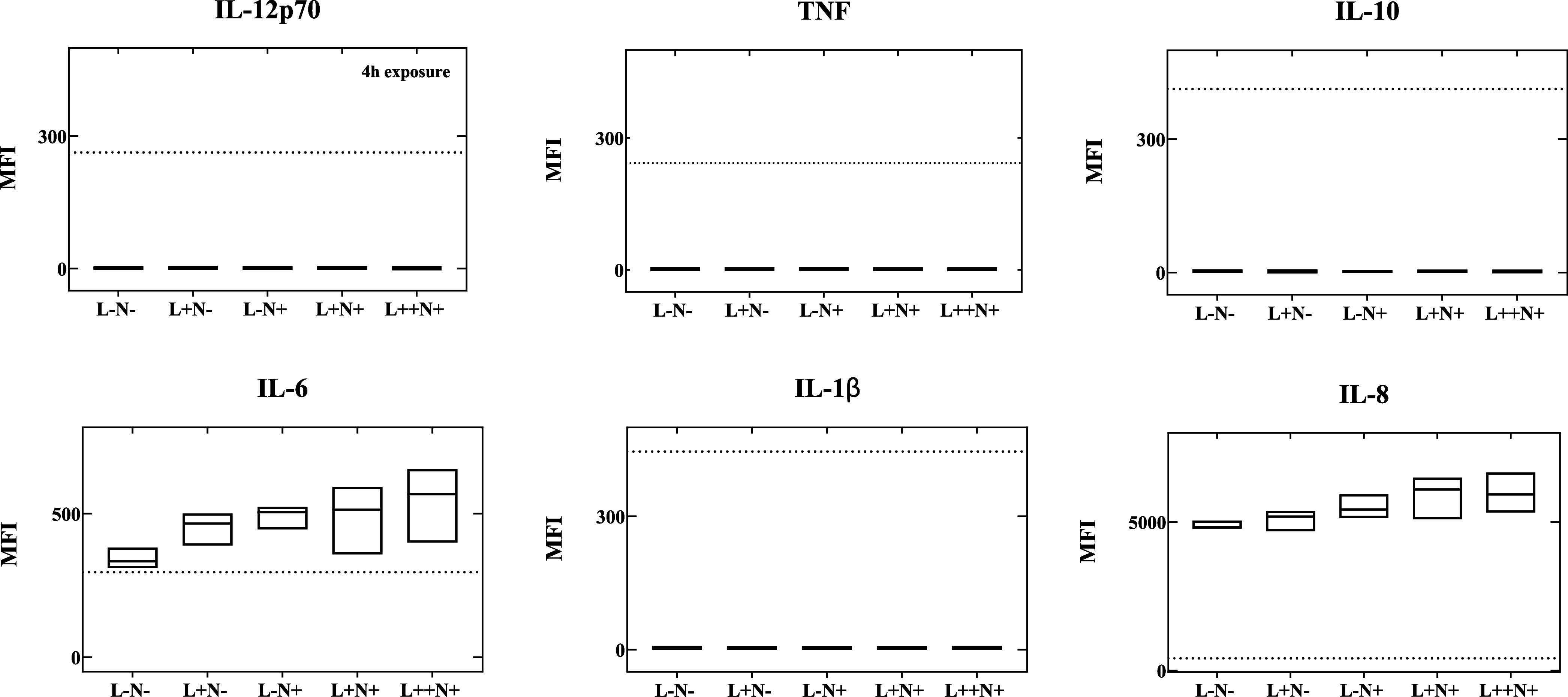
Inflammatory profile of the 3D alveolar epithelial model after a 3 h NP exposure and collection of the basal medium 4 h after exposure, assessed using the CBA human inflammatory cytokines kit. Abbreviations: L– (no laser), L+ (laser 43 °C), L++ (laser 47 °C), N– (no NP), N+ (NP at 10 mg/mL), MFI (mean fluorescence intensity). Dashed line: detection limit.

Complementary studies were also performed to investigate the levels of inflammatory cytokines after these exposures. In this assay, flow cytometric evaluation of the 3D alveolar epithelium model did not reveal substantial elevations in the biomarkers evaluated. However, in terms of IL-6 and IL-8 expression 4 h after laser associated with NP exposure, notable increases of 58% (L++N+) and 22%, (L++N+), respectively, were observed in these particular biomarkers compared to the control group (Figure 6). Although statistical significance was not achieved, an apparent upward trend was observed in these biomarkers with a higher thermal dose.

IL-6 serves as a cytokine marker indicative of immune system activation and is often upregulated in cancer cells. It plays a crucial role in mediating B-cell differentiation and T-cell activation, growth, and differentiation.^39^ Its multifaceted activities include an anti-inflammatory function at low doses, inducing an M2-like state in macrophages, while at higher doses, it can exhibit pro-inflammatory responses, particularly in reaction to tissue injury.^40^ The observed increase in IL-6 expression with higher thermal doses may align with the tissue viability results illustrated in Figure 3e.

In contrast, IL-8, also known as CXCL8, acts as a powerful chemotactic factor, stimulating neutrophil and monocyte/macrophage migration to the sites of inflammation. However, it also functions as a potent angiogenic factor depending on its expression levels.^41^ In our investigation, its expression remained relatively low, suggesting a potential role in attracting immune cells to the site of thermally mediated tissue injury, which could contribute to the promotion of an abscopal effect.

After thermal-therapy-induced cell death, the release of NPs and tumor neoantigens is expected. Macrophages recruited to the region can internalize both NPs and tumor antigens. Iron oxide-based NPs are well-documented to induce M1 polarization,^7^ with M1 macrophages serving as antigen-presenting cells that could activate the immune system, thus exerting control over tumor growth and metastasis. The optimal outcome would involve robust activation of the immune response via a thermally induced abscopal effect. Some evidence from murine models suggests the plausibility of this phenomenon.^34,35^ Future efforts aim to test this hypothesis by employing a more intricate 3D reconstructed alveolar A549 lung cancer model, incorporating various cell types in an air–liquid interface system.

Finally, it might be relevant to note that the thermal dose is obtained through thermal camera measurements, but this technology is known to determine only the tissue surface temperature.^42,43^ Since we showed that most NPs are internalized in the cells and aggregated inside vesicles, one anticipates that the thermal dose expressed by this type of measurement might be revealing a lower thermal dose than the real local intracellular one.^44,45^ To deal with this issue, one needs to develop thermal sensors that are efficient in distinct environments and that are not influenced by NP agglomeration. This is beyond the scope of this study, but we hope that in the future, we can check this more carefully using a nanothermometry strategy.^46−48^

## 4 Conclusions

In this study, we evaluated the potential of Zn- and Co-doped magnetite NPs for thermal therapy using a human alveolar 3D lung cancer model. The results indicate that cobalt increases the saturation magnetization and magnetic anisotropy of the NP, which, for the conditions evaluated, resulted in a better magnetic hyperthermia response in comparison to magnetite NPs of similar size. The low Co content maintained the biocompatibility of the NP, i.e., we found no evidence of toxicity up to high particle concentration. Photothermal therapy showed better heat generation compared with magnetic hyperthermia under the experimental conditions investigated. For this reason, a detailed PTT study was performed in a 3D alveolar reconstructed lung cancer tissue model. To the best of our knowledge, this is the first report of this type of research in the literature. The use of the human tissue model might help future clinical translation since it reflects a better real human scenario. We found a decrease in tissue viability with a higher thermal dose (higher temperatures). Additionally, increased intracellular ROS levels, reduced mitochondrial activity, and elevated active caspase-3 levels indicate apoptosis as the primary mechanism of cell death. Inflammatory cytokines were also evaluated. NPs and PTT did not influence TNF, IL-10, IL-1B, and IL-12p70. In contrast, IL-6 and IL-8 were detected, and the median release value was found to increase with higher thermal dose. The effect correlates with tissue injury, suggesting the potential for a heat-triggered immunological response by activating and attracting immune cells to thermally mediated sites of tissue injury sites. In summary, the study underscores the promising prospect of using photothermal therapy responses with ZnCo-doped magnetite NPs to induce an immune-mediated antitumor effect in cancer therapy.

## Supporting Information Available

Original EDS data showing internalized NPs by cells; trypan blue exclusion assay of A549 cells after exposure to different concentrations of NP; binary image analysis of converted tissue microscopic images (PDF)
