# Supplementary material for: Human 3D Lung Cancer Tissue Photothermal Therapy Using Zn- and Co-Doped Magnetite Nanoparticles
Source: ACS Biomater Sci Eng. 2025 Jan 24;11(2):1084–95. doi: 10.1021/acsbiomaterials.4c01901 (PMC11815621; doi:10.1021/acsbiomaterials.4c01901)
Supplement: Supplementary file 1 — ab4c01901_si_001.pdf [file ab4c01901_si_001.pdf]

Supplementary Information: Human 3D Lung Cancer Tissue Photothermal Therapy using Zn and Co Doped Magnetite Nanoparticles

Edynara Cruz de Moraes,[1] Marcella Miranda Siqueira Furtuoso Rodrigues,[2] Rafaela Campos de Menezes,[2] Marcus Vinícius-Araújo,[1] Marize Campos Valadares,[2,*] and Andris Figueiroa Bakuzis[1,3,†]

[1]*Institute of Physics, Federal University of Goiás, Goiânia, Goiás, 74690-900, Brazil*
[2]*ToxIn—Laboratory of Education and Research in In Vitro Toxicology, Federal University of Goiás, Goiânia 74690-631, Brazil*
[3]*CNanoMed, Federal University of Goiás, Goiânia, Goiás, 74690-631, Brazil*

(Dated: October 11, 2024)

S1. ORIGINAL ENERGY DISPERSIVE X-RAY SPECTROSCOPY (EDS) DATA

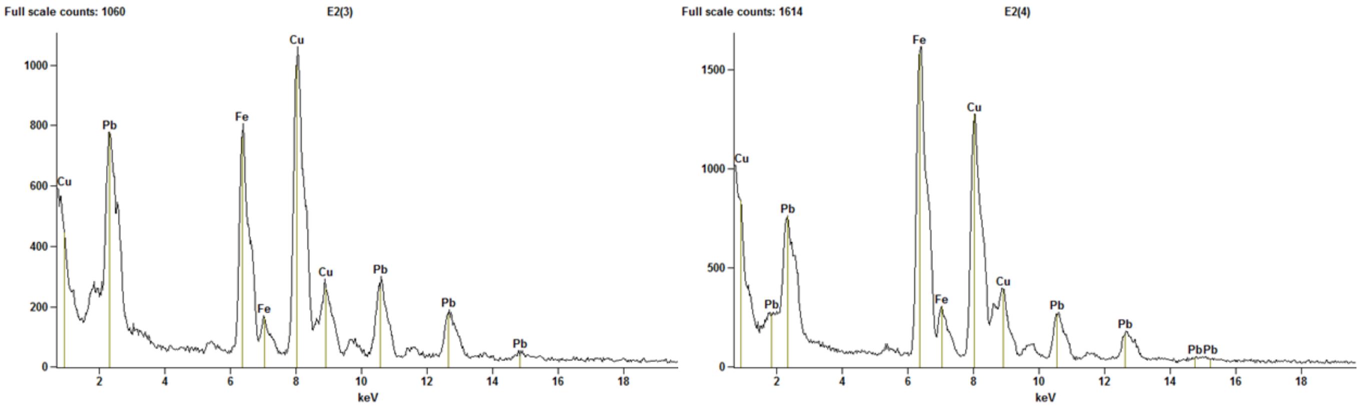

(a) (b)

FIG. S1: EDS from region (a) 1 and (b) 2 presented in figure 3.

* marizecv@ufg.br
† bakuzis@ufg.br

S2. TRYPAN BLUE EXCLUSION ASSAY (TBE)

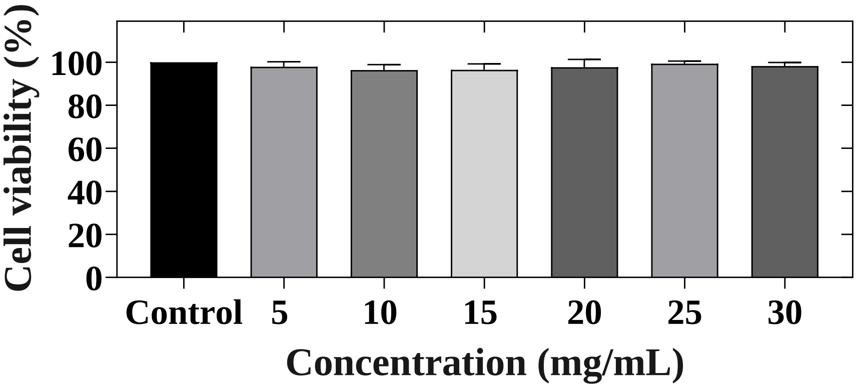

FIG. S2: The effects on a monolayer of A549 cells after exposure to different concentrations of NP were assessed by the Trypan blue exclusion assay (TBE) after 24 hours;

S3. BINARY IMAGES ANALYSIS

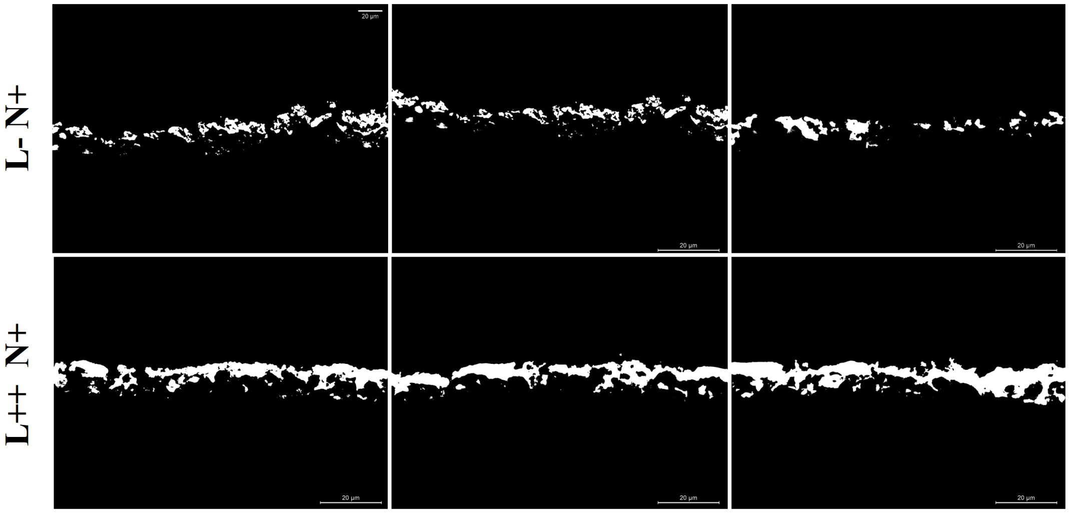

FIG. S3: Converted tissue microscopic images to grayscale and thresholding for segmentation of nanoparticles from the cellular layer using ImageJ software.

Using ImageJ software, the images were converted to grayscale (Image → Type → 8 bits) to facilitate segmentation; see Figure (S3). Then a threshold was applied to segment the nanoparticles from the background and the cellular layer (Image → Adjust → Threshold). The images were then transformed into binary (Process → Binary → Make Binary). The nanoparticles layer was defined as a Region of Interest (ROI) using the free-hand selection tool. With ROI selected, the average pixel intensity corresponding to the nanoparticles embedded in the tissue was measured (Analyze → Measure), see Figure (S4). This procedure was repeated for three images of each of the L- N+ and L++ N+ groups to allow comparison.

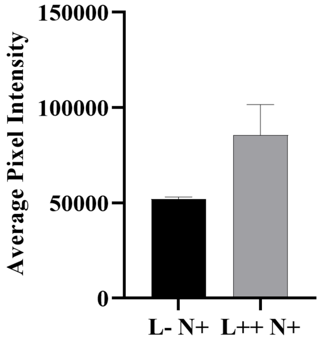

FIG. S4: Average pixel intensity of nanoparticles in the tissue image ROI.
